# Production of L-Theanine Using *Escherichia coli* Whole-Cell Overexpressing γ-Glutamylmethylamide Synthetase with Baker’s Yeast

**DOI:** 10.4014/jmb.1910.10044

**Published:** 2020-02-25

**Authors:** Soo-Yeon Yang, Yeong-Hoon Han, Ye-Lim Park, Jun-Young Park, So-young No, Daham Jeong, Saerom Park, Hyung Yeon Park, Wooseong Kim, Seung-Oh Seo, Yung-Hun Yang

**Affiliations:** 1 Department of Biological Engineering, College of Engineering, Konkuk University, Seoul 05029, Republic of Korea; 2 Department of Bioscience and Biotechnology, Konkuk University, Seoul 0509, Republic of Korea; 3 College of Pharmacy and Graduate School of Pharmaceutical Sciences, Ewha Womans University Seoul 0760, Republic of Korea; 4 Department of Food Science and Nutrition, The Catholic University of Korea, Bucheon 1662, Republic of Korea; 5 Institute for Ubiquitous Information Technology and Applications, Konkuk University, Seoul 0029, Republic of Korea

**Keywords:** L-theanine, whole-cell biocatalyst, baker’s yeast, ATP regeneration

## Abstract

L-Theanine, found in green tea leaves has been shown to positively affect immunity and relaxation in humans. There have been many attempts to produce L-theanine through enzymatic synthesis to overcome the limitations of traditional methods. Among the many genes coding for enzymes in the L-theanine biosynthesis, glutamylmethylamide synthetase (GMAS) exhibits the greatest possibility of producing large amounts of production. Thus, GMAS from *Methylovorus mays* No. 9 was overexpressed in several strains including vectors with different copy numbers. BW25113(DE3) cells containing the pET24ma::*gmas* was selected for strains. The optimal temperature, pH, and metal ion concentration were 50oC, 7, and 5 mM MnCl_2_, respectively. Additionally, ATP was found to be an important factor for producing high concentration of L-theanine so several strains were tested during the reaction for ATP regeneration. Baker’s yeast was found to decrease the demand for ATP most effectively. Addition of potassium phosphate source was demonstrated by producing 4-fold higher L-theanine. To enhance the conversion yield, GMAS was additionally overexpressed in the system. A maximum of 198 mM L-theanine was produced with 16.5 mmol/l/h productivity. The whole-cell reaction involving GMAS has greatest potential for scale-up production of L-theanine.

## Introduction

Tea is widely consumed worldwide because of its delicate flavor and physiological benefits because it contains many components such as catechins, caffeine, vitamins, L-theanine, and mineral elements [1]. Among them, L-theanine, also known as γ-glutamylethylamide, is naturally found in Japanese green tea leaves and contributes to the quality of green tea [2]. The physiological activities of L-theanine include effects as remarkable relaxation in humans, enhancing immunity, and decreasing anti-oxidative stress and diseases [3]. Accordingly, the demand for L-theanine is increasing annually and large scale-production methods are necessary to supply L-theanine to the food and pharmaceutical industry [4].

Many methods for the synthesis of L-theanine have been investigated extensively such as extraction from tea leaves [5, 6], chemical synthesis [7], and enzymatic synthesis [8, 9]. Direct extraction has numerous limitations for large-scale production such as its high cost, time-consuming steps, and demanding operational processes. Additionally, the chemically synthesized product is a racemic mixture of the L-form and D-form, thereby reducing product purity and requiring additional purification. [10]. Thus, biological synthesis of L-theanine has attracted much attention recently.

The enzymes involved in L-theanine production include γ-glutamyltranspeptidase (GGT), L-glutaminase, L-glutamine synthetase (GS), or γ-glutamylmethylamide synthetase (GMAS). Under overexpression of GGT in *E. coli* followed by purification, 156 mM L-theanine was produced using glutamine and ethylamine as substrates [11]. Additionally, production of 126 mM L-theanine was achieved using purified L-glutaminase from *Pseudomonas nitroreducens* through a γ-glutamyl transfer reaction of glutamine [12]. However, conversion yields remain low because GGT and L-glutaminase tend to hydrolyze L-glutamine to other γ-glutamyl derivatives [13]. Liu *et al*. produced 254 mM L-theanine using GMAS from *Methylovorus mays* No. 9 strain by glutamate and ethylamine through the hydrolysis of ATP. Compared to other enzymes, GMAS shows greater potential for producing high concentrations of L-theanine [14].

Many synthetases require high ATP concentrations during catalysis to ensure high titers of various products [15, 16]. Therefore, systems for ATP regeneration have been investigated [17-19]. These methods use a variety of enzymes capable of synthesizing ATP, such as polyphosphate kinase, acetate kinase, and pyruvate kinase [20, 21]. However, the use of pyruvate kinase and acetate kinase is limited by the high cost of phosphoryl donors and their product inhibition activity. Additionally, both systems possess broad substrate specificities, which can regenerate other types of nucleosides and 2’-deoxynucleoside triphosphates, such as GTP, UTP, and CTP [22]. The system using polyphosphate kinase shows low activity towards specific substrates, so that it causes poor availabilities. As an alternative to ATP regeneration, the use of baker’s yeast has been considered in many studies [23-25]. Sugar-fermentative systems can effectively produce ATP as an energy source from AMP or ADP, effectively [26]. Therefore, the excessive consumption of ATP can be overcome by taking advantage of the energy transfer system of baker’s yeast in an ATP-dependent reaction [27, 28].

In this study, we developed a whole-cell biotransformation system for production of L-theanine. GMAS from *M. mays* No. 9 was overexpressed in *E. coli* BW25113 and was used for the synthesis of L-theanine using glutamate and ethylamine as substrates. Compared to conventional usage of purified enzymes, this whole-cell system can mitigate complex purification processes and decrease operational costs to enhance economic feasibility. Through this result, the cell cultivation and reaction conditions were optimized to achieve a high titer of L-theanine. Additionally, we found that ATP and different phosphate sources affected the reaction. To our knowledge, this is the first application of whole-cell overexpressed by GMAS for L-theanine production.

## Materials and Methods

### Reagents

Sodium L-glutamate monohydrate (>99%) and ethylamine hydrochloride (>98%) were purchased from Sigma-Aldrich Co. (USA). L-Theanine (>98%) and Adenosine 5’-triphosphate disodium salt hydrate were obtained from Tokyo Chemical Industry Co. (Japan). Baker’s yeast was purchased from ACH Food Companies, Inc. (Oakbrook Terrace, USA). Tris hydrochloride (>99%), Tris-base (>99.9%), sodium borate decahydrate (>99%), isopropyl-β-d-thiogalactopyranoside (>99%), and other medium components were obtained from Biosesang Co. (Korea). Diethyl ethoxymethylenemalonate (DEEMM) was purchased from Fluka Co. (Japan) for the derivatization reaction.

### Bacterial Strains, Plasmids, and Media

*E. coli* DH5α strain was used as the host for gene cloning. Based on the sequence of GMAS from *Methylovorus mays* No. 9, GMAS was codon-optimized and synthesized by Cosmogenetech (Korea) and utilized as the PCR template for gene amplification. Amplified genes were inserted into pET24ma (constructed by Dr. David Sourdive, Pasteur Institute, France) containing a p15A replication origin, pXMJ19, and pMAL vectors using restriction enzymes and T4-ligase. The constructed plasmid was transformed into competent *E. coli* cells to prepare the whole-cell biocatalyst [29]. All bacterial strains and plasmids are provided in **Table 1**. All *E. coli* strains were pre-cultured in 5 ml lysogeny broth (LB) medium containing 10 g/l tryptone, 5 g/l yeast extract, and 5 g/l sodium chloride with antibiotics by inoculating a single colony from an agar plate. Cells were cultured in a shaking incubator (Han‐Beak Science Co., Korea) at 37°C with 200 rpm. The pre-culture was inoculated into 50 ml of main culture medium containing antibiotics in a 250-ml baffled Erlenmeyer flask and incubated at 37°C with shaking [30, 31]. Upon reaching an OD600 of 0.6–0.7, the cultures were induced by adding isopropyl β-d-1-thiogalactopyranoside (IPTG) at 0.5 mM. The cultures were then incubated at 25°C with shaking for 16 h. Cultures were harvested by centrifugation at 5,500 rpm for 10 min at 4°C, and the cell pellet was washed with deionized water [32]. The cell suspension was stored at 4oC until further use and referred to as whole-cell.

### Whole-Cell Reaction

GMAS activity was evaluated using the whole-cell as a catalyst. The reaction was performed with 100 mM glutamate, 100 mM ethylamine, 200 mM glucose, 60 mM ATP, 5 mM MnCl_2_, 10 mM MgCl_2_, 300 mM potassium phosphate and 40 mg/ml baker’s yeast. Each parameter was varied to identify the overall optimal reaction conditions. The initial pH was adjusted to 7 with 5 N NaOH. Each parameter was varied to identify the optimal reaction conditions. The reaction solution was incubated at 50oC for 24 h in a shaking mixer at 1,200 rpm. The reaction was stopped by heating at 90°C for 5 min. The reaction solution was then diluted to a suitable concentration for high-performance liquid chromatography (HPLC, YL-9100; Korea) analysis [33].

### Derivatization and HPLC Analysis

Amine derivatives were prepared in a mixture of 300 μl borate buffer (50 mM, pH 9), 100 μl methanol, 47 μl distilled water, 50 μl target sample, and 3 μl DEEMM [34]. The derivatization reaction was performed at 70oC for 2 h to derivatize glutamate, L-theanine, and ethylamine. Analysis by HPLC was performed after derivatization of the reaction products at a UV-absorbance of 284 nm. Chromatographic separation was conducted using a reverse-phase C18 column (ZORBAX SB-C18 column, 4.6 × 250 mm, 5 μm particle size; Agilent Technologies, USA) and the column temperature was maintained at 35°C. The mobile phase was composed of 100% acetonitrile (solvent A) and 25 mM sodium acetate buffer pH 4.8 (solvent B). The flow rate was maintained at 1 ml/min and the composition of solvent A to B (A:B, v/v) was changed with the following gradient program: 0 min (20:80), 2 min (25:75), 32 min (60:40), 37 min (20:80), 40 min (20:80).

## Results and Discussion

### Optimization of Protein Expression Conditions and Cultivation Medium

**Fig. 1:**
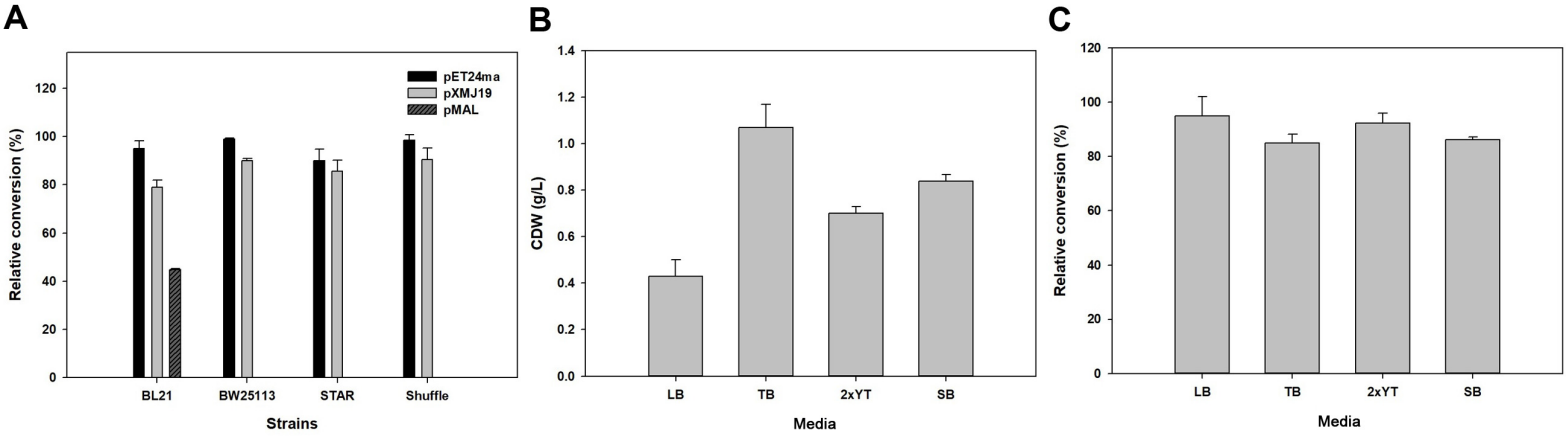
Optimization of expression and cultivation conditions. GMAS activities were compared in several expression strains containing vectors with different copy numbers for producing L-theanine (**A**). Cultivation medium were tested by comparing cell dry weight (**B**) and relative conversion of substrates (**C**). The results were indicated as relative conversion, which regards the highest value as 100% conversion.

To obtain a high level of protein expression, GMAS activities in the whole-cell reaction were investigated using various vectors with different copy numbers in several *E. coli* strains (Fig. 1A). pET24ma (copy numbers 10–12), pXMJ19 (copy number 10-20), and pMAL (copy number 20) were evaluated as GMAS overexpression vectors. The results were indicated as relative conversion, which regards the highest value as 100% conversion. Results showed that all strains containing the pET24ma::*gmas* exhibited the highest activities. The pMAL::*gmas* only exhibited activity in the BL21 strain. When comparing GMAS overexpression using pET24ma and pXMJ19, GMAS overexpression with pET24ma showed slightly higher activities in all expression strains. Additionally, evaluation of the pCDF::*gmas*, pACYC::*gmas*, and pRSF::*gmas*, revealed no activities. To explain these results, SDS-PAGE was performed to see GMAS expression level. However, the expression levels from the three kinds of vectors were much lower than those from vectors that showed activity (data not shown). Therefore, we decided to use BW25113 harboring pET24ma::*gmas* in further experiments.

After selecting the expression vector and strains, we tested various expression media because it can affect cell growth and expression levels [35]. Several media were compared, such as terrific broth (TB), 2xYT, and super broth (SB), which contain higher nitrogen levels than LB media, which is commonly used for *E. coli* cultivation. The results revealed that TB showed the greatest influence on cell growth after 24 h of culture (Fig. 1B). The cell dry weight (CDW) by TB was the 2.5, 1.6 and 1.3-fold higher than those grown in LB, 2xYT, and SB, respectively. However, the cell activity after cultivation in TB was slightly lower than that in LB and 2xYT (Fig. 1C). However, it is inefficient to use cells with a low growth rate but slightly higher activities from an industrial perspective. Therefore, TB was utilized to prepare the biocatalyst.

### Optimization of Whole-Cell Reaction with Temperature, pH, and Metal ions

**Fig. 2:**
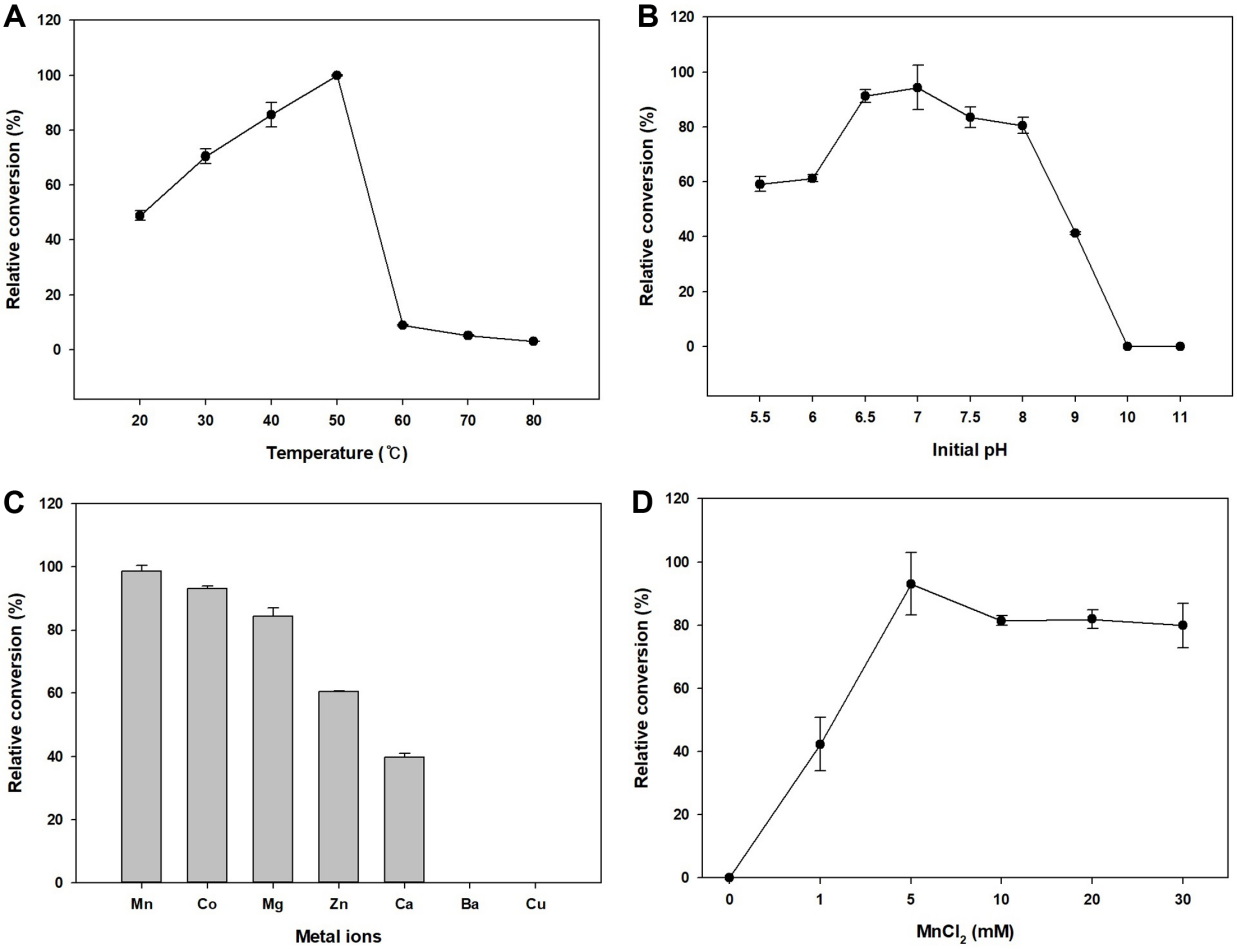
Optimization of whole-cell reaction conditions. Optimal temperature was evaluated from 20°C to 80°C (**A**) and optimal initial pH was evaluated from 5.5 to 11 (**B**). Various metal ions were investigated (**C**) and optimal MnCl_2_ was found (**D**).

To increase the conversion yields of the whole-cell reaction, reaction conditions considered as key factors were optimized. Reaction temperatures ranging from 20–80°C were tested to determine the optimal temperature (Fig. 2A). The highest activity was observed at 50°C, which differs from a previous report that showed an optimal temperature of 37°C [14]. This may be because our study used a whole-cell as biocatalyst rather than purified enzyme. A whole-cell bioconversion system generally possesses advantages such as robustness against harsh reaction conditions and higher stability and storability than purified enzymes [36]. The optimal pH was also studied to enhance production of L-theanine (Fig. 2B). Various initial pH values were tested from 5.5 to 11, which were adjusted by adding Tris-HCl buffer and NaOH solution. The activity increased gradually until pH 7. However, the activity started to decrease from pH 7 and no activity observed at pH 10 and 11. In further experiments, the reaction temperature and pH were adjusted to 50°C and 7, respectively, as the optimal conditions.

In the ATP-dependent reactions, metal ions are often necessary as cofactors for the main enzyme for effective synthesis of ATP [37, 38]. Thus, many types of metal ions were investigated to identify the most effective factor during the whole-cell reaction (Fig. 2C). Among these metal ions, Mn^2+^, Co^2+^, Mg^2+^, Zn^2+^, and Ca^2+^ were found to influence the reaction. Particularly, the reaction with Mn^2+^ could produce 2.4-fold higher L-theanine than that with Ca^2+^. However, activities were not observed when Ba^2+^ and Cu^2+^ were utilized. To determine the optimal concentration of Mn^2+^, MnCl_2_ was fed into the reactant to the desired concentration (Fig. 2D). As a result, the reaction containing 5 mM MnCl_2_ could produce the largest amount of L-theanine. Additionally, 10 mM MgCl_2_ had positive effect in L-theanine production when we fixed ATP and MnCl_2_ concentrations (Fig. S1). Thus, 5 mM MnCl_2_ and 10mM MgCl_2_ were used in further experiments.

### Effect of Co-Factors on Reaction

**Fig. 3:**
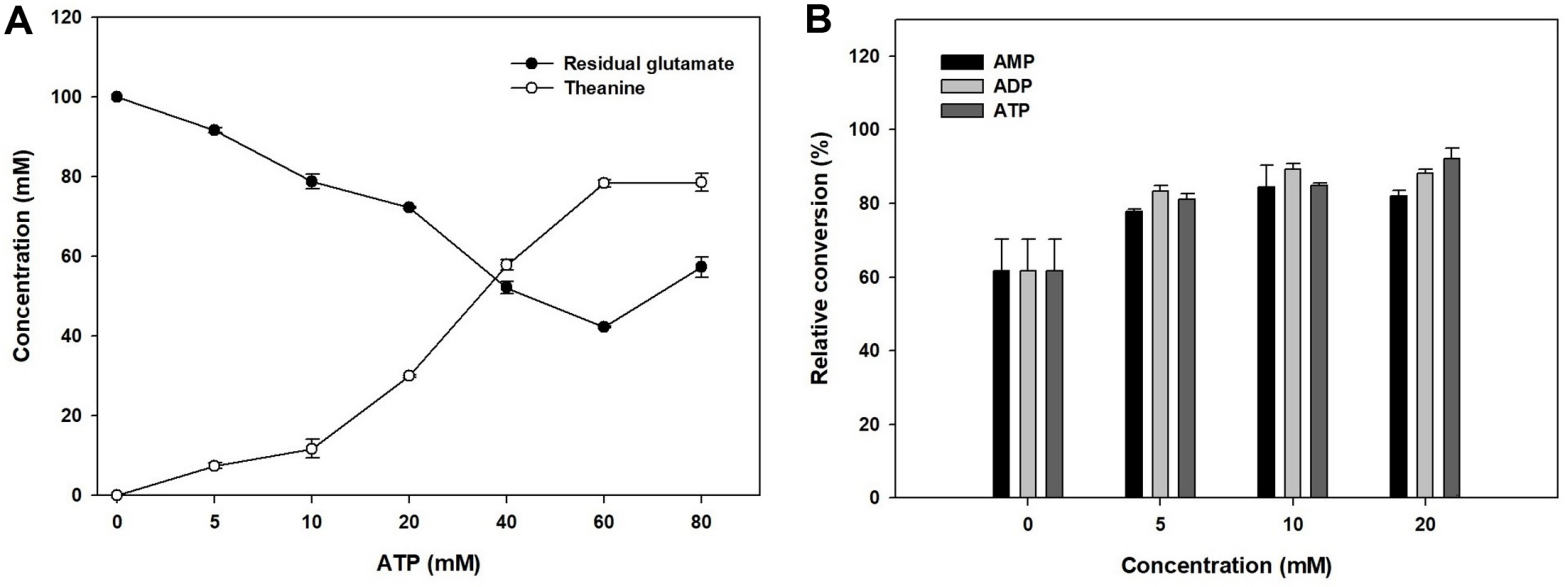
The effect of different ATP concentration on the production of L-theanine by the GMAS enzyme. Optimal ATP concentration was determined by detecting glutamate and L-theanine (A) and the effects of ATP, ADP, and AMP. All components were tested at various concentrations (B).

To confirm the effects of ATP on the GMAS enzyme, different concentrations of ATP were utilized for L-theanine production (Fig. 3A). The substrate level was 100 mM and pH was pH 7. No conversion was observed in the absence of ATP and glutamate also remained as initial substrates. As the concentration increased, L-theanine was also increased gradually. Approximately 80 mM L-theanine was achieved with 80% conversion when more than 60 mM ATP was used. These data indicate that GMAS is an ATP-dependent enzyme.

We also tested other kinds of energy sources such as ADP and AMP for comparison with ATP in L-theanine production (Fig. 3B). AMP or ADP can be used as energy source because we introduced an ATP regeneration system. Therefore, the source most effective for L-theanine production was evaluated. When these factors were compared to the control which contained no sources of ATP, the conversion yields were greater than control in all parameters. The use of AMP resulted in lower conversion yields than the other sources at all concentrations. Thus, we used ATP considering its lower cost, although ADP and ATP showed similar conversion yields.

### Effects of Baker’s Yeast and Phosphate for ATP Regeneration

**Fig. 4:**
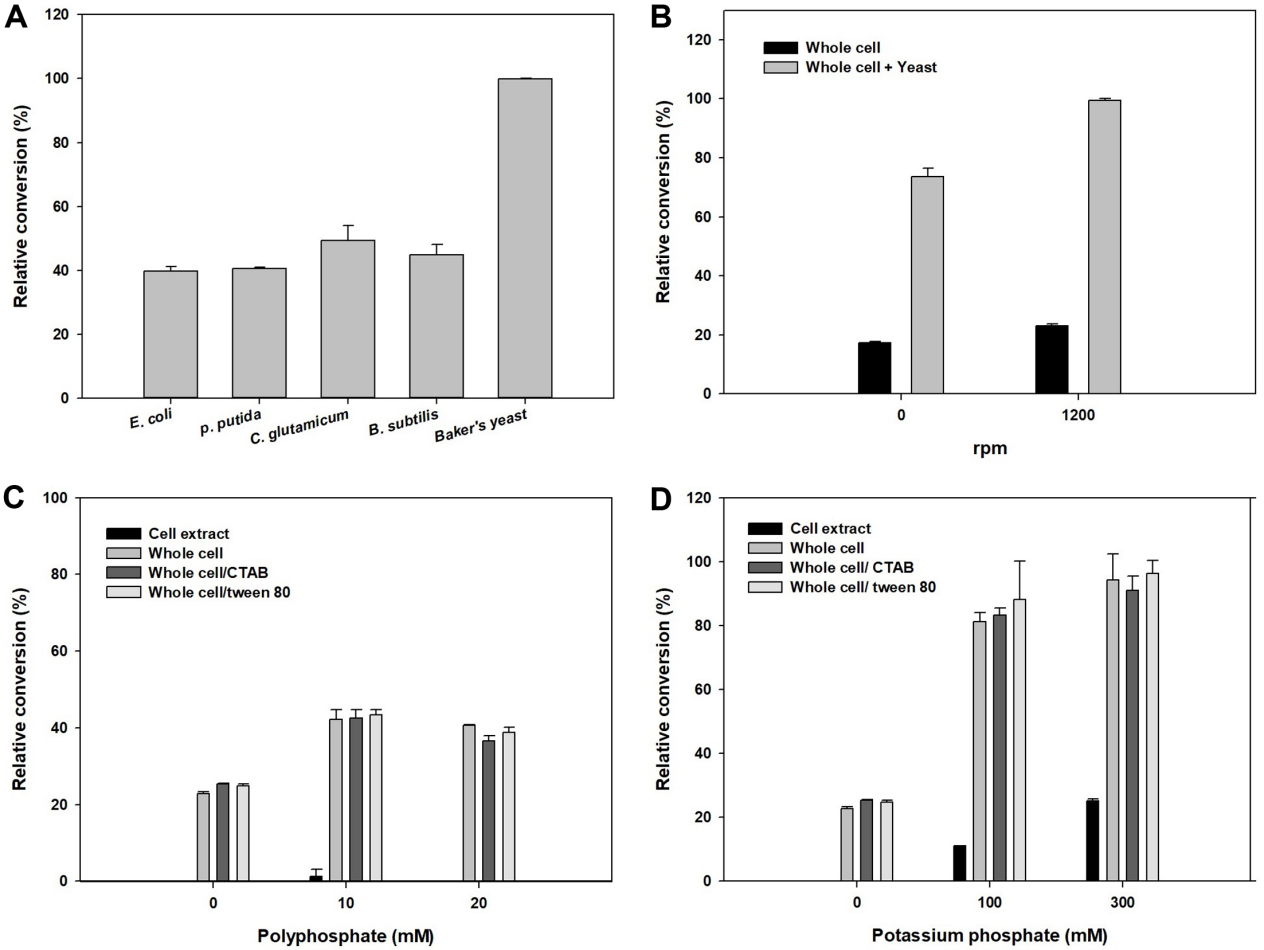
Effect of baker’s yeast for ATP regeneration. Various strains were cultivated during the reactions for ATP regeneration (**A**). Activities were checked in comparison between control reaction and reaction containing baker’s yeast depending on rpm rates (**B**). The effects of phosphate sources were examined by *E. coli* whole-cell and baker’s yeast using polyphosphate (**C**) and potassium phosphate (**D**).

Various strains were cultivated with the whole-cell reaction for energy transfer (Fig. 4A). Then, we added 200 mM glucose for cell growth. Among them, baker’s yeast was the most effective for ATP synthesis, which leaded to the highest production of L-theanine. The relative conversion of *E. coli*, *Pseudomonas putida*, and *Bacillus subtilis* were nearly half of that from baker’s yeast. *Corynebacterium glutamicum* showed slightly higher than those of activity with 53% relative conversion.

To clarify the effects of baker’s yeast on ATP regeneration, whole-cell reactions were compared after adding baker’s yeast (Fig. 4B). Approximately 3.8-fold higher L-theanine levels were obtained in the static reaction. When we increased the reaction speed to 1,200 rpm, 4.3-fold higher L-theanine was produced, which was more efficient than in the static reaction. Thus, ATP regeneration shows better results when the baker’s yeast is added for respiration than for fermentation.

In the reaction using sugar fermentative methods with baker’s yeast, phosphate sources are necessary to enhance production. Therefore, polyphosphate and potassium phosphate were tested (Figs. 4C and 4D). Additionally, some treatments were carried out to evaluate cell permeability, which facilitates the transport of substrates and product. Cetyl trimethyl ammonium bromide (CTAB), polyoxyethylene sorbitan monooleate (Tween 80), and sonicated whole-cell (cell extract) were compared to the whole-cell system. Among them, the cell extract showed low L-theanine production. Additionally, activities using the whole-cell were similar to those of the whole-cell treated with CTAB and Tween 80. However, polyphosphate slightly influenced conversion yields compared to the control, which showed a 1.8-fold higher value in the presence of 10 mM polyphosphate than the control. To enhance the titers, additional phosphatases are needed to convert the polymers into monomers. In contrast, potassium phosphate was greatly effective for increased the production of L-theanine. Conversion yields were approximately 4-fold higher than those of the control in the presence of 300 mM potassium phosphate. When we tested sodium phosphate, L-theanine production was lower than that after potassium phosphate addition (Fig. S2).

### Enhanced L-Theanine Production by Repetitive Use and Additional GMAS Expression

**Fig. 5:**
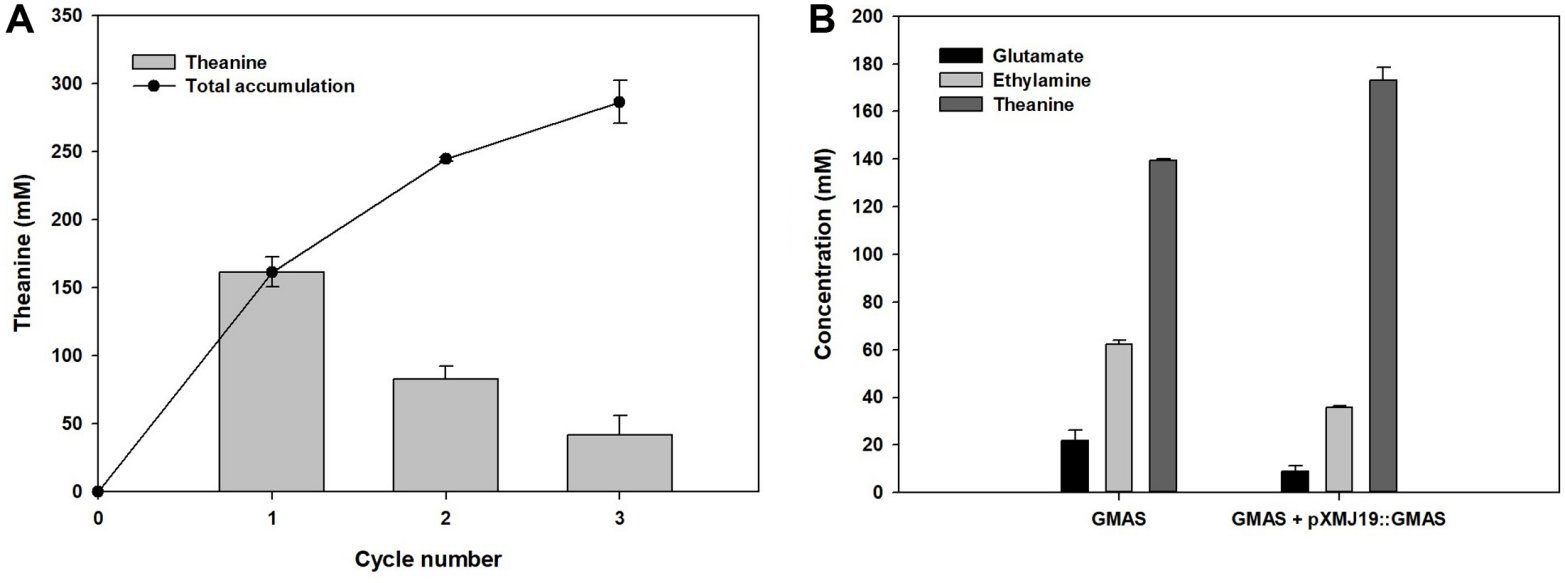
Enhanced production by repeating GMAS whole-cell for accumulation of L-theanine (A) and overexpressing additional *gmas* gene (B).

To examine the reusability of GMAS enzyme for the repetitive use of the whole-cell, the cell was recycled 3 times (Fig. 5A). The L-theanine concentration after each cycle and the accumulated L-theanine were monitored. The reactants containing cells were centrifuged after 24 h, and then the reacted medium was replaced with fresh substrate medium. After the first cycle, 161 mM L-theanine was recorded from 200 mM substrates. The produced L-theanine concentration was decreased by 0.51- and 0.26-fold in the second and third cycles, respectively. The total amount of L-theanine was obtained until 286.3 mM in 3 cycles.

Cells harboring pXMJ19::*gmas* exhibited activities as high as those of BW25113 (DE3) harboring pET24ma::*gmas* according to previous data and the repetitive use of the whole-cells. Therefore, the additional vector was inserted into BW25113 (DE3) harboring pET24ma::*gmas*. When the substrate concentration was 200 mM, approximately 140 mM L-theanine was obtained with 70% conversion (Fig. 5B). After pXMJ19::*gmas* was introduced, production increased to 178 mM with 89% conversion while substrate consumption also increased. Accordingly, we found that additional GMAS expression affects positively to enhance L-theanine production.

**Fig. 6:**
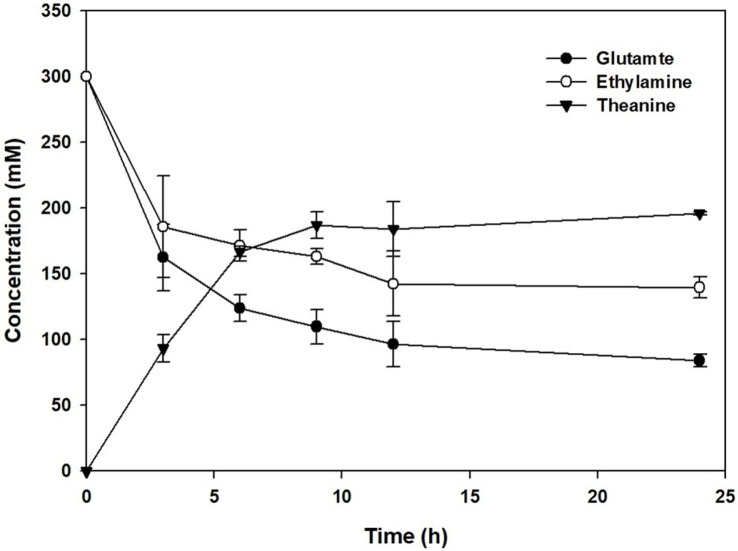
L-theanine production monitoring by time-dependent manner under optimized concentration of substrates and co-factor.

Finally, the time-dependence of the reaction was carried out with BW25113 (DE3) overexpressing pXMJ19::*gmas* and pET24ma::*gmas*. The reaction was performed with 300 mM glutamate, 300 mM ethylamine, 200 mM glucose, 60 mM ATP, 5 mM MnCl_2_, 10 mM MgCl_2_, 300 mM potassium phosphate and 40 mg/ml baker’s yeast (Fig. 6). Because most substrates were converted to L-theanine in the previous data (Fig. 5B), we increased the substrate level to 300 mM. Glutamate and ethylamine as the substrates started to be consumed after 3 h. After the reaction was complete, approximately 100 mM glutamate and 148 mM ethylamine remained. 198 mM L-theanine could be achieved within 12 h We conclude L-theanine can be produced by 16.5 mmol/l/h productivity.

Many studies have been conducted to evaluate the use of various enzymes for L-theanine production. L-Theanine exhibits effects such as anti-tumor, relaxation, and enhanced immunity. Previous reports show that among the enzymes used in L-theanine synthesis, GMAS shows potential for application in industrial production. However, the process of GMAS enzyme purification is difficult, costly, and time-consuming. In contrast, the whole-cell is easy to handle and does not require complicated procedures for its preparation. Therefore, the whole-cell reaction involving GMAS has greatest potential for scale-up production of L-theanine.

To construct a whole-cell system for L-theanine, protein expression and the reaction conditions were optimized. Additionally, the effect of baker’s yeast in ATP regeneration was clarified. Additional expression was also efficient for enhancing L-theanine production. To our knowledge, this is the first application of a whole-cell reaction of GMAS to develop an L-theanine production system.

## Supplemental Materials

Supplementary data for this paper are available on-line only at http://jmb.or.kr.

## Figures and Tables

**Table 1:** List of bacterial strains and plasmids.

Strain/plasmid	Relevant information	Reference
Bacterial strains		
*E. coli* DH5α	F^-^ φ 80*lacZ* M15 *endA recA* hsdR(r_k_^-^m_k_^-^) *supE thi gyrA relA* Δ (*lacZYAargF*)U169	[39]
*E. coli* BL21(DE3)	F^-^ *ompT* hsdSB(rB^-^mB^-^) *gal dcm*	Novagen
*E. coli* BW25113	F' λ^-^ Δ(*araD-araB*)567, Δ*lacZ*4787(::rrnB-3), lambda^-^, *rph*-1, Δ(*rhaD_rhaB*)568, hsdR514	CGSC
*E. coli* BW25113(DE3)	λDE3 lysogen of BW25113	This study
*E. coli* Shuffle	*fhuA2 lacZ*::T7 gene1 [*lon*] *ompT* *ahpC gal* λatt::pNEB3-r1cDsbC (Spec^r^, lacIq) Δ*trxB sulA*11 R(mcr-73::miniTn10– TetS)2 [dcm] R(zgb-210::Tn10 --TetS) *endA1* Δ*gor* Δ(*mcrC-mrr*)114::IS10	[40]
*E. coli* Star ^TM^ (DE3)	F^-^ *ompT* *rne*131 *lon* hsdSB (rB^-^mB^-^) *gal dcm* λ(DE3)	Invitrogen
*B. subtilis* str. 168		ATCC
*C. glutamicum* ATCC13032		ATCC
*P. putida* KT2440		ATCC
Baker’s yeast		
Plasmid		
pET24ma	Km^r^.1 MCS site with T7 promoter, *lac* operator, RBS. P15A replicon. Copy number: 10–12	[41]
pCDF duet 1	Spec^r^.2 MCS site with T7 promoter, *lac* operator, RBS. CloDF13 replicon. Copy number: 20–40	Novagen
pRSF duet 1	Km^r^.2 MCS site with T7 promoter, *lac* operator, RBS. RSF1030 replicon (NTP1). Copy number: >100	Novagen
pACYC duet 1	Cm^r^.2 MCS site with T7 promoter, *lac* operator, RBS. P15A replicon. Copy number: 10–12	Novagen
pMAL	Amp^r^. MCS site with tac promoter, *lac* operator, RBS, ColE1 replicon. Copy number: 20	Novagen
pXMJ19	Cm^r^. MCS site with tac promoter, *lac* operator, pBL1 and pUC replicon	[42]
pET24ma::*gmas*	*gmas* of *Methylovorus mays* No. 9 inserted in pET24ma	This study
pCDF duet 1::*gmas*	*gmas* of *Methylovorus mays* No. 9 inserted in pCDF duet 1	This study
pRSF duet 1::*gmas*	*gmas* of *Methylovorus mays* No. 9 inserted in pRSF duet 1	This study
pACYC duet 1::*gmas*	*gmas* of *Methylovorus mays* No. 9 inserted in pACYC	This study
pMAL::*gmas*	*gmas* of *Methylovorus mays* No. 9 inserted in pMAL	This study
pXMJ19::*gmas*	*gmas* of *Methylovorus mays* No. 9 inserted in pXMJ19	This study
