# Supplementary material for: Production of L-Theanine Using Escherichia coli Whole-Cell Overexpressing γ-Glutamylmethylamide Synthetase with Baker’s Yeast
Source: J Microbiol Biotechnol. 2020 Feb 25;30(5):785–92. doi: 10.4014/jmb.1910.10044 (PMC9728304; doi:10.4014/jmb.1910.10044)
Supplement: Supplementary file 1 [file JMB-30-5-785-supple.pdf]

J. Microbiol. Biotechnol.
https://doi.org/10.4014/jmb.1910.10044

Supplementary data

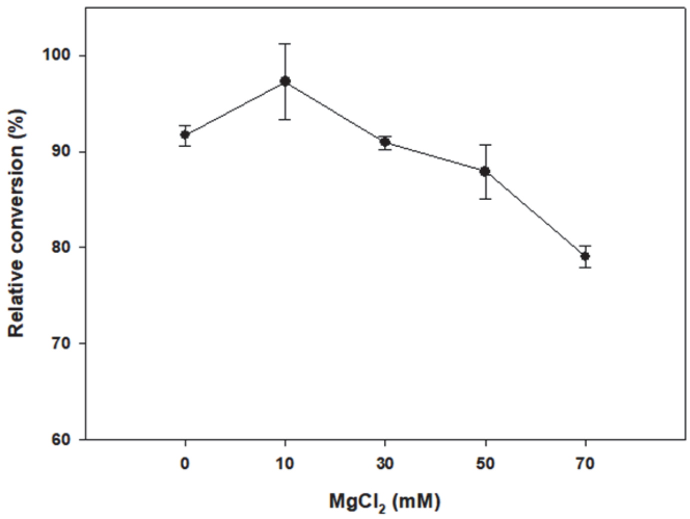

Fig S1. Relative conversion depending on $MgCl_2$ concentration.

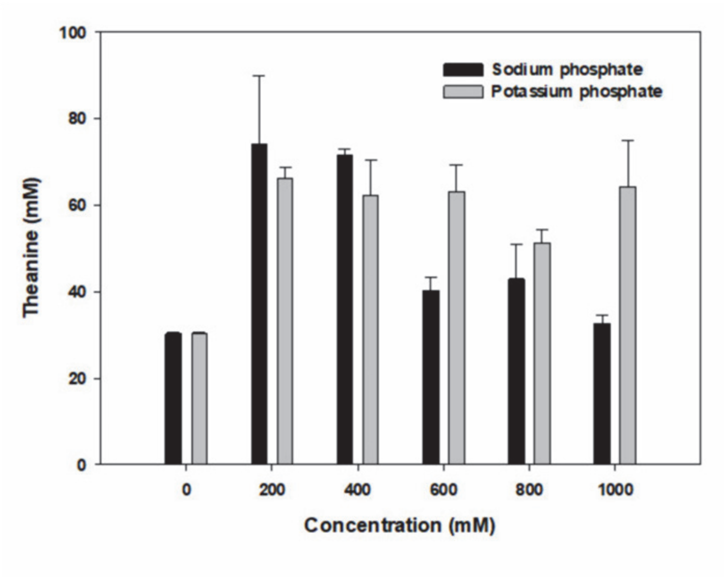

Fig S2. Effect of sodium phosphate and potassium phosphate at various concentration.
